# Anticancer Activity of Demethylincisterol A3 and Related Incisterol-Type Fungal Products

**DOI:** 10.3390/life15101638

**Published:** 2025-10-21

**Authors:** Christian Bailly

**Affiliations:** 1 Institut de Chimie Pharmaceutique Albert Lespagnol (ICPAL), Faculté de Pharmacie, University of Lille, 59000 Lille, France; christian.bailly@univ-lille.fr; 2 UMR9020-U1277-CANTHER-Cancer Heterogeneity Plasticity and Resistance to Therapies, CHU Lille, CNRS, Inserm, OncoLille Institute, University of Lille, 59000 Lille, France; 3 OncoWitan, Scientific Consulting Office, 59290 Lille, France

**Keywords:** anticancer, degraded steroids, demethylincisterol A3, fungi, incisterol, volemolide

## Abstract

Highly degraded sterols belonging to the incisterol group have been identified in a large set of microorganisms. The leading product in the family is demethylincisterol A3 (DM-A3), isolated from various fungi and endowed with marked antitumor properties. Since the initial discovery of incisterol from a marine sponge in the 1990s, more than 30 incisterol-type natural products have been identified, essentially from fungi. An overview of these products, their bio-origin, chemical synthesis, and associated pharmacological properties is presented. The series includes diverse incisterol and demethylincisterol derivatives, chaxines, volemolide, different analogues (salimyxins, phellinignincisterols, daedatrin D, inonotoide F, aplykurodinone-1, dendrodoristerol), and a glycoside derivative (xyloneside), all bearing a tetracyclic incisterol framework. An analysis of the anticancer mechanism of the action of DM-A3 underlined the three main components of its activity associated with the (i) inhibition of β-catenin and the Wnt signaling pathway, (ii) inhibition of tyrosine phosphatase SHP2 (IC_50_ = 6.75 µM) implicated in cancer cell survival and differentiation, and (iii) blockade of α7nAchR activation coupled with inhibition of acetylcholinesterase (IC_50_ = 11.16 µM). A comprehensive picture of the DM-A3 mechanism of action is discussed, highlighting the uniqueness of the compound as a dual SHP2/AchE inhibitor able to attenuate an inflammatory response through the cholinergic anti-inflammatory pathway. The review shed light on this little-known category of incisterol-type natural products, with the objective of promoting further research into this neglected group of anticancer agents.

## 1. Introduction

Sterols are essential molecules largely present in mammalian species (e.g., cholesterol), plants (phytosterols), fungi (ergosterol), and eukaryotes in general, but relatively rare in bacteria. In humans, they serve as membrane structural elements to regulate the fluidity of biological membranes and as precursors to steroid hormones, bile acids, and other bioactive molecules. Sterols exert many roles in the human body, and their metabolism is tightly regulated. Dysfunction in the metabolism and transport of cholesterol, sterol intermediates, and derivatives (notably oxysterols) plays an important role in various pathologies, such as metabolic diseases and cancers [1].

Whatever their origin, sterols are largely investigated owing to their therapeutic interest and their use as food supplements [2]. In particular, the therapeutic potential of phytosterols to treat inflammatory diseases is increasingly recognized [3]. Major efforts are deployed to better understand the biological functions of sterols and their intermediates which can be exploited in the agricultural and pharmaceutical industries [4]. Naturally occurring sterols, modified sterols (metabolites), as well as synthetic sterol derivatives, are considered for drug design or as biomarkers [5,6].

**Figure 1:**
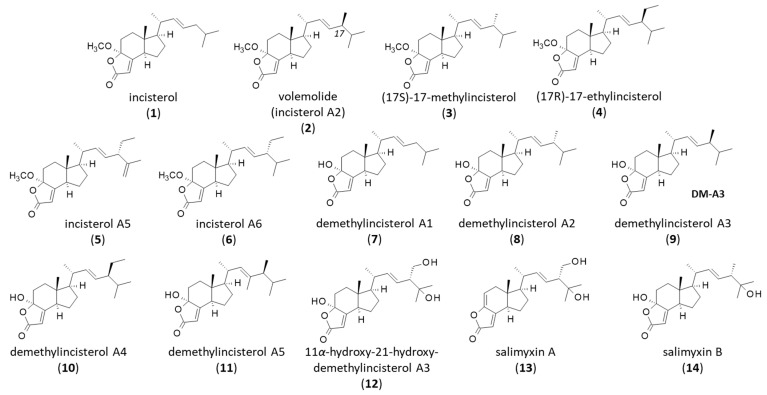
Incisterol and related natural products.

Among the many sterol derivatives, there is a category of products often referred to as “highly degraded sterols” corresponding to unusual sterols with a modified skeleton, usually produced by microorganisms. The microbial degradation and bioconversion of cholesterol and other sterols lead to the production of bioactive derivatives [7,8]. The aerobic degradation of the sterol tetracyclic nucleus by microorganisms, often associated with a structural rearrangement, can lead to the production of a large diversity of derivatives of biological interest [9,10]. Notably, the microbial transformation of sterols can afford a group of highly degraded sterols related to the tricyclic molecule incisterol (Figure 1). This tricyclic scaffold, initially discovered from the marine sponge *Dictyonella incisa* [11], has been found in several natural products endowed with interesting bioactivities.

The present article provides an overview of all incisterol-type terpenoids and an analysis of their pharmacological properties. The objective is to better delineate the incisterol products family, highlighting the diversity of natural products in this group and identifying key properties of the most interesting compounds. The study aimed to comprehensively analyze the products and their origin, and to point out the advantages and limitations of the lead compound demethylincisterol A3 (DM-A3). The overall goal is to promote further research into this atypical series of natural products.

## 2. Incisterol and Demethylincisterol Derivatives

Four incisterols were initially isolated from the Mediterranean sponge *Dictyonella incisa*, namely incisterol (**1**), (17*R*)-17-methylincisterol (also known as volemolide) (**2**), (17*S*)-17-methylincisterol (**3**), and (17*R*)-17-ethylincisterol (**4**) (Figure 1). They are believed to originate from the degradation of C-27/C-29 sterols, notably an oxidation of the sterol B-ring [11]. Chianese et al. referred to incisterol A2 for (17*S*)-17-methylincisterol (**3**), incisterol A5 (**5**) for (17*R*)-17-ethylincisterol and incisterol A6 (**6**) for the related compound with a 17-ethyl-15,18-diunsaturated side chain (Figure 1). Remarkably, incisterols A5 and A6 were shown to induce transactivation of the pregnane X receptor (PXR) upon binding to the ligand-binding domain (LBD) of PXR. They behaved as PXR agonists, stimulating mRNA expression of the two PXR target genes *CYP3A4* and *MDR1* [12].

The related compound demethylincisterols A1, A2, A3, and A4 (**7**–**10**) were subsequently isolated from the marine sponge *Homaxinella* sp. collected in Korea. Demethylincisterol A3 has been referred to as (17*R*)-4-hydroxy-17-methylincisterol, found in the edible mushroom *Agrocybe chaxingu*, but it is the same compound [13]. *A. chaxingu* is largely cultivated in China and used as a medicinal product. Apparently, demethylincisterols would originate from the enzymatic degradation of the tetracyclic precursor 5R,8R-epidioxy sterol by symbiotic microbes [14]. The series has been completed in recent years with the discovery of demethylincisterol A5 (**11**) from a rice fermentation culture of the endophytic fungus *Aspergillus tubingensis* YP-2. This latter compound has revealed modest antiproliferative activities against cultured cancer cells (IC_50_ = 11.05 and 19.15 μM against A549 and HepG2 cells, respectively), and its close analogue demethylincisterol A3 (**9**) was a little more potent (IC_50_ = 5.34 and 12.03 μM, respectively) [15].

The most interesting product in the series is demethylincisterol A3 (**9**, hereafter designated DM-A3) which has been found in different species, notably in the mushrooms *Daedaleopsis confragosa* [16] and *Penicillium brocae* G2131 [17], the sponge-derived fungus *Gymnascella dankaliensis* [18], from fruiting bodies of the mushrooms *Tricholoma imbricatum* [19] and *Agaricus blazei* [20], the fungus *Xylaria allantoidea* SWUF76 [21], and from other species. The compound is largely present in fungi. An analysis using the mass spectrometry search engine MASST pointed out about 60 fungal species that contain DM-A3 [16]. In addition, derivatives of DM-A3 have been found in some species. This is the case for 11*α*-hydroxy-21-hydroxy-demethylincisterol A3 (**12**) isolated together with DM-A3 from the cultured mycelia of *Ganoderma capense* [22]. All species producing DM-A3 and related products are listed in Table 1.

DM-A3 has revealed potent cytotoxic activity to HeLa, A549, and HepG2 cells with IC_50_ values in the nM range [37]. However, in another study, the same compound showed a more modest cytotoxic activity against cancer cells, with IC_50_ of 26.49 and 28.45 μM toward HCT116 and HeLa cells at 72 h, respectively [21]. The discrepancy has not been explained, but the difference may be due to a cytostatic effect observed in the first case (24 h of cell treatment) vs. a cytotoxic effect in the second case (72 h). Cytotoxicity (IC_50_) in the range of 5–12 μM has been reported in other studies (at 72 h) [15]. *Agaricus blazei* is an edible mushroom and a medicinal species with a rich profile in secondary metabolites [45,46]. It is a producer of DM-A3 [23] and the analogue volemolide (**2**) discussed below [24]. The anticancer properties of DM-A3 have been confirmed in another study aimed at evidencing the capacity of the product to induce cell cycle perturbation (G1 arrest), to trigger apoptosis in cancer cells, and to reduce tumor cell migration and invasion. The drug was shown to inhibit the Wnt signaling pathway and transcription of the *β-catenin* gene in cancer cells. A direct binding of DM-A3 to the β-catenin receptor was proposed on the basis of a molecular modeling analysis. The drug would bind to a small cavity on β-catenin, establishing two H-bonds with residue Thr-433 of the protein. This hypothesis has not been validated experimentally at present, but the conjecture is plausible considering the demonstrated capacity of other sterols to interfere with β-catenin [47].

Interestingly, a marked antitumor activity has been evidenced in mice bearing HeLa or HepG2 tumors upon treatment with DM-A3. The drug was administered i.v. at 0.1 mg/kg reduced the tumor growth, without inducing toxic effects [48]. The anticancer potential of DM-A3 has been evidenced in other studies, notably when the product was combined with the standard antimetabolite drug 5-fluorouracil (5-FU) to suppress the growth of cervical cancer cells. In this case, the two drugs synergized to repress tumor cell growth, and the activity was dependent on the expression of phosphatase SHP2, considered as a potential target for DM-A3 [49]. DM-A3 selectively inhibited the enzyme SHP2, much more potently than the related phosphatase SHP1 (IC_50_ = 6.75 and 57.78 µM, respectively) and showed no effects on other phosphatases like Cdc25b and PTB1B. It is a non-competitive inhibitor of SHP2, blocking the interaction of the enzyme with the pathway element GRB2-associated-binding protein 1 (Gab1) which is a broad-range kinase regulator and a key enzyme to modulate resistance and sensitivity to antitumor therapies [50,51]. Drugs targeting SHP2 are increasingly considered for the treatment of solid tumors, notably colon cancers [52,53]. They are also investigated to combat other pathologies, such as neurodegenerative diseases [54]. The selective targeting of the oncogenic tyrosine phosphatase SHP2 with DM-A3 is thus important to guide the development of analogues. A comparison of the SHP2 binding capacity of all incisterol derivatives would be useful.

The product 4-hydroxy-17-methylincisterol (DM-A3) has been reported to inhibit DNA polymerase α (pol. α) with a potency comparable to that observed with the two analogues 17-methylincisterol and 4-acetyl-17-methylincisterol [55]. The former entity, analogue to chaxine A (see below), is an interesting natural product with immunomodulatory properties. It has been shown to suppress the expression and production of cytokines interleukin-2 (IL-2), IL-4, tumor necrosis factor-α (TNF-α), and interferon-γ (IFN-γ) in phytohemagglutinin-stimulated human peripheral blood mononuclear cells (PBMC). 4-Hydroxy-17-methylincisterol also reduced PBMC proliferation and inhibited Ca^2+^ mobilization in these cells [23]. This natural product has been isolated from diverse fungi, including *Trichoderma asperellum* SCNU-F0048 [56], *Agrocybe chaxingu* [13], *Harposporium anguillulae* [29], and the endolichenic fungus *Pleosporales* sp. [57]. In the medicinal mushroom *Amauroderma amoiensis* (= *A. rugosum*), (17R)-17-methylincisterol (**2**) has been isolated and found to weakly inhibit acetylcholinesterase (46.3% inhibition of AchE at 50 µM) [25].

**Figure 2:**
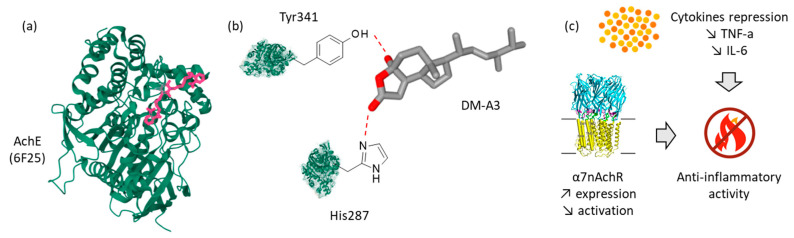
A molecular docking analysis has suggested that demethylincisterol A3 (DM-A3) can bind to the active site of acetylcholinesterase (AchE), via contacts with residues Tyr341 and His287 [27]. (**a**) The AchE protein and its ligand C35 (PDB: 6F25) and (**b**) interaction of AchE with DM-A3 from the fungus *Aspergillus tennesseensis* 1022LEF, associated with (**c**) inhibition of cytokine expression and increased expression of the alpha-7 subunit of the human nicotinic acetylcholine receptor (α7nAchR) and repression of the activation of its downstream signaling pathways [27].

A much more potent inhibition of AchE was reported more recently with demethylincisterol A3 (DM-A3). In this case, the compound was shown to dose-dependently inhibit the enzyme (IC_50_ = 11.16 µM) and to reduce production of both TNF-α and IL-6 in LPS-activated RAW 246.7 macrophages. A docking analysis suggested a direct binding of DM-A3 to the AchE enzyme, via specific contacts with the key residues Tyr341 and His287 near the enzyme active site [27] (Figure 2). There is a narrow and deep gorge in the AchE enzyme, well adapted to accommodate extended small molecules. Residues Tyr341 and Trp286 (adjacent to His287) play an important role in drug binding to AchE [58,59]. The exact mode of binding of DM-A3 to AchE remains hypothetical at present (based on a molecular docking analysis), but the capacity of the product to inhibit the enzyme has been validated experimentally [27].

Remarkably, DM-A3 exhibited a potent anti-inflammatory activity and was able to up-regulate expression of the alpha-7 subunit of the human nicotinic acetylcholine receptor (α7nAchR) which is a neurotransmitter receptor with an immunoregulatory function. The authors suggested that the inhibitory effects of DM-A3 on LPS-induced inflammation were partly dependent on α7nAchR activation, resulting in a downstream inhibition of the phosphorylation of transcription factors NF-κB and Stat3 [27]. α7nAchR is a key element of the cholinergic anti-inflammatory pathway. It is important to identify and develop α7nAchR-targeting small molecules with immunoregulatory functions [60]. It would be interesting to compare the immunoregulatory and anti-inflammatory properties of all demethylincisterol derivatives, notably DM-A2 (**8**) which has revealed a capacity to inhibit expression of cyclooxygenase-2 (COX-2) and cytosolic prostaglandin E2 synthase (cPGES) in LPS-activated RAW 246.7 macrophages [61].

An interesting study has evidenced the capacity of DM-A3 to inhibit quorum sensing (QS) and biofilm formation in the pathogenic bacteria *Bacillus cereus*. QS is a bacterial density-related communication mechanism that mediates the synthesis of virulence factors and biofilm formation. DM-A3 has been shown to reduce biofilm formation of *B. cereus* (by 50% at 3.12 µg/mL), associated with a decrease in protease and hemolysin production. The product also markedly repressed expression of several genes implicated in QS, thereby weakening the pathogenicity of the *bacillus* species [39].

## 3. Salimyxins

Salimyxins A and B (**13**,**14**) are two rare structural analogues of DM-A3 discovered from the marine cholesterol-producing myxobacterium *Enhygromyxa salina*. Salimyxin A (**13**) presents a C3-C4 double bond in the tricyclic nucleus. They bear one or two hydroxyl groups at C16/C19 (Figure 1). They both revealed little or no antibacterial activity. Only salimyxin B (**14**) showed a marginal antimicrobial activity toward *Micrococcus luteus* (MIC = 32 µg/mL) [62]. Salimyxin B has also been identified from the medicinal plant-derived fungus *Aspergillus* sp. TJ23, and in this case, a modest cytotoxic effect toward HepG2 cells was reported (IC_50_ = 9.87 µM) [63]. The cytotoxicity level of salimyxin B is consistent with that observed with DM-A3.

## 4. Chaxines

**Figure 3:**
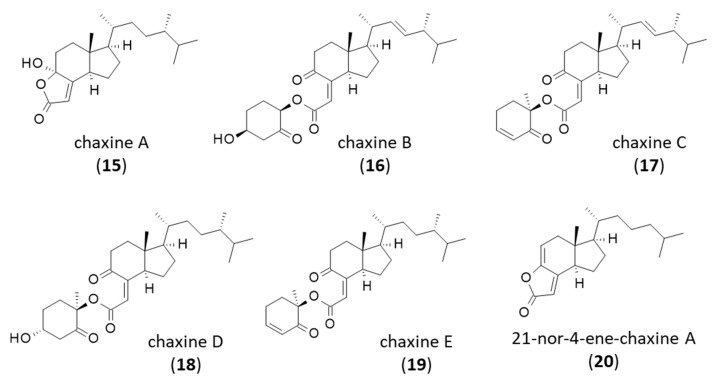
Structures of chaxines.

There are five compounds in the series. The first one, chaxine A (**15**), was described in 2006 from the edible mushroom *Agrocybe chaxingu* from which it was co-isolated together with (17*R*)-4-hydroxy-17-methylincisterol (DM-A3). The two compounds **9** and **15** were found to inhibit osteoclast formation by about 50% at the test dose of 4.8 µM [13]. The property is interesting to combat bone diseases such as postmenopausal osteoporosis. The four other chaxines B-E (**16**–**19**) isolated from the same fungus are structurally distinct, with a bicyclic, not a tricyclic core [64,65] (Figure 3). Chaxines B and C may represent biosynthetic intermediates to DM-A3 [66]. Chaxine C has also been isolated from an *Aspergillus* sp. fungus together with (4S,17R)-4-hydroxy-17-methylincisterol [28]. Chaxine C (**17**) apparently exhibits a marked cytotoxic activity toward MCF7 breast cancer cells comparable to that of DM-A3 (IC_50_ = 10.2 and 10.9 µM, respectively), and it is more potent against A-549 lung carcinoma cells (IC_50_ = 7.9 and 27.7 µM, respectively) [19].

A derivative of chaxine A, designated 21-nor-4-ene-chaxine A (**20**), has been isolated recently from the South China sea sponge *Spongia officinalis* [67]. Chaxine B (**16**) has been found in *Ganoderma lucidum* spores’ oil [68]. Chaxine C is the most potent compound in the series, with marked cytotoxic properties apparently superior to those of DM-A3. It inhibited the proliferation of different cancer cell lines (HeLa, HCT116, MCF-7, HT29) much more potently than DM-A3 [21]. These chaxine products may be of interest to combat cancer, but their mechanism of action warrants further investigation. A chemical route to the total synthesis of both chaxine A (**15**) and DM-A3 (**9**) from ergocalciferol has been reported, enabling the preparation of analogues and a better definition of structure-activity relationships in this series [69].

## 5. Volemolide

**Scheme 1:**
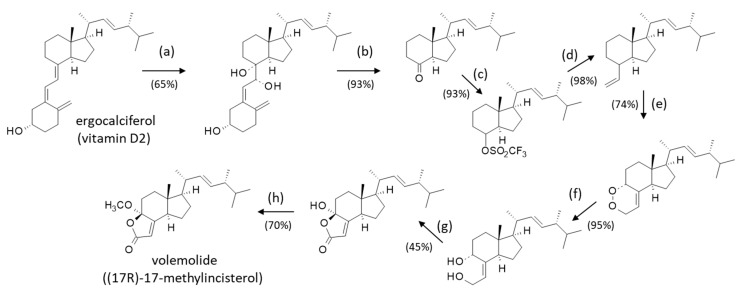
Synthesis of volemolide from ergocalciferol (vit. D2) [73]. (a) KMnO_4_, EtOH, −20 °C, 2 h. (b) Pb(OAc)_4_, CH_2_Cl_2_, −15 °C, 30 min. (c) NaN(SiMe_3_)_2_, THF, −78 °C for 15 min with addition of phenyl-triflimide and then 0 °C 3 h. (d) Vinyl(tributyltin (Bu_3_SnCH=CH_2_), Pd(PPh_3_)_4_, LiCl, THF, reflux 1 h. (e) Singlet oxygen (^1^O_2_), 5,10,15,20-tetraphenyl-21H,23H-porphine (TPP), CH_2_Cl_2_, 300 W lamp, −78 °C, 3 h. (f) Nodium bis(2-methoxyethoxy)aluminum hydride (Red Al), toluene, r.t. 1 h. (g) CrO_3_, H_2_SO_4_, H_2_O/acetone, 0 °C, 20 min. (h) HCl, MeOH, r.t., 12 h. See [73] for more details.

Volemolide ((17*R*)-17-methylincisterol) (**2**) is a norsterol derivative initially isolated from the edible mushroom *Lactarius volemus* [70]. It was later found in a few other fungi, such as *Tricholoma imbricatum* [19], *Harposporium anguillulae* [32], *Penicillium herquei* [35], and *Diaporthe* sp. LG23 [31]. It has also been found in the endophytic fungus *Aspergillus versicolor* isolated from the marine green alga *Codium fragile* [71]. The compound presents a marked cytotoxicity profile, comparable to that of chaxine C (**17**) [19]. Volemolide has been shown to inhibit the growth of *Candida albicans* [24] and to present an activity against the parasite *Trypanosoma brucei* with an efficacy comparable to the standard drug suramin (IC_50_ = 1.41 and 1.58 μg/mL, respectively) without noticeable cytotoxic effects [72]. The antitrypanosomal activity of incisterol derivatives has been barely studied thus far; it deserves further investigation. Many years ago, Sodano and coworkers realized the first total synthesis of (17*R*)-17-methylincisterol (volemolide) starting from ergocalciferol (vitamin D2) in eight steps with an overall yield of 13% [73] (Scheme 1). This type of synthesis should facilitate the design of analogues.

## 6. Other Derivatives

**Figure 4:**
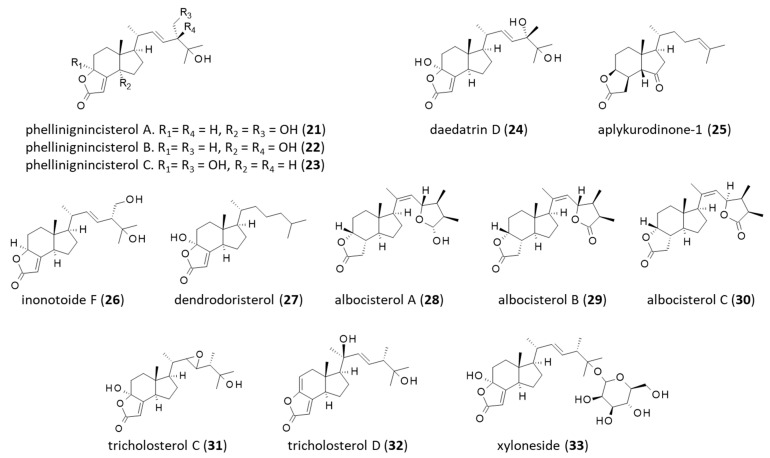
Other incisterol-type natural products.

Thirteen additional rare products structurally related to incisterol have been identified. The first three are phellinignincisterols A-B-C (**21**–**23**), isolated from the fungus *Phellinus igniarius* [74] (Figure 4). They bear a structural analogy with the products (i) daedatrin D (**24**) isolated from the Chinese wood-rotting fungus *Daedaleopsis tricolor* [75], (ii) aplykurodinone-1 (**25**) from the skin of the marine anaspidean *Syphonota geographica* [76], (iii) inonotoide F (**26**) from the medicinal fungus *Inonotus obliquus* [77], (iv) dendrodoristerol (**27**) from the nudibranch mollusk *Dendrodoris fumata* [78], (v) albocisterols A-C (**28**–**30**) from the Basidiomycete *Antrodiella albocinnamomea* [79], and (vi) tricholosterols C-D (**31**,**32**) isolated from the fruiting bodies of *Tricholoma terreum* [80]. The latter compound, tricholosterol D, has been shown to inhibit NO production in LPS-activated RAW264.7 macrophages (Figure 4). These natural products are all incisterol derivatives.

Finally, there exists a unique glycoside derivative of incisterol designated as xyloneside A (**33**). The β-D-mannopyranose moiety is attached to the terminal -OH of the side chain incisterol [81]. This natural product (**33**), isolated from ascomycetous species *Xylaria hypoxylon* FB, is arguably the only glycosylated compound known in this family at present.

## 7. Conclusions

There are many types of “highly degraded steroids” comprising a tricyclic or bicyclic core, often isolated from marine or terrestrial species [82,83]. The chemical diversity reflects the broad range of steroid-degrading organisms, notably among bacteria and fungi [84]. A focus on the incisterol skeleton led to the identification of 33 natural products belonging to a few subgroups, in particular demethylincisterol derivatives, chaxines, volemolide, and a handful of other derivatives. Altogether, these products have been isolated or identified from about 50 fungal species. They represent a structurally homogeneous group of natural products, possibly with a common pharmacological profile.

A major natural product emerges from this incisterol panel, DM-A3, which has revealed interesting anticancer properties. The product has shown cytotoxic properties toward different cancer cell lines in vitro and a synergistic action when combined with the standard anticancer drug 5-FU against cervical cancer cells [49]. The drug showed a remarkable anticancer efficacy in vivo, at least when administered i.p., reducing significantly tumor growth (from HepG2 and Hela xenografts) in mice [48]. DM-A3 warrants further investigation as an anticancer agent. However, prior to testing the compounds in other tumor xenograft models, it would be essential to investigate their pharmacokinetic properties (including their oral bioavailability) and to determine their toxicity profile. It is an important prerequisite prior to expanding in vivo studies.

The mechanism of action of DM-A3 has been delineated, at least in part, with three lines of action: (i) repression of *β-catenin* transcription with inhibition of the Wnt signaling pathway, (ii) selective inhibition of tyrosine phosphatase SHP2 (IC_50_ = 6.75 µM) and blockade of the Gab1 pathway implicated in cell differentiation and survival signaling, and (iii) inhibition of α7nAchR activation coupled with a binding to and inhibition of acetylcholinesterase (IC_50_ = 11.16 µM) [27,48,49]. This multimodal mechanism of action translates into a capacity to restrict tumor cell growth, differentiation, and a remodeling of the immune microenvironment. This latter aspect warrants further investigations, but, nevertheless, the mechanism of action is of prime interest in combating resistant tumors. There is a constant medical need for novel options to combat chemo-resistant cancers.

The dual action of DM-A3 as an inhibitor of SHP2 and AchE is innovative. It is not unique (a similar dual SHP2/AchE inhibitory action has been reported with the polyphenol ellagic acid [85]), but it represents a novel option to explore. Here again, it will be important to investigate the safety of the product first because SHP2 is one of the phosphatases that regulate acetylcholine receptor cluster formation in muscle cells [86,87]. The relationship between AChE and SHP2 needs to be better defined. DM-A3 offers an option to help dissect this potential relationship.

Thus far, studies in this series have been essentially concerned with the discovery of incisterol-type products from fungi and with the mechanism of action of the lead product DM-A3. The time has come to consider all naturally occurring incisterol derivatives such as the 33 compounds reported here, and to implement chemical strategies to design analogues, as done for volemolide, for example. Hopefully, the present incisterol panorama will encourage the discovery and design of new products.

## Figures and Tables

**Table 1:** Fungi known to produce demethylincisterol A3 or derivatives.

Species	Observations	References
*Agaricus blazei* Murill	Isolation of a few metabolites including DM-A3 and volemolide from this terrestrial basidiomycete.	[20,23,24]
*Amauroderma amoiensis* (= *A. rugosum*)	Identification of 14 metabolites including DM-A3 and (17*R*)-17-methylincisterol. Modest inhibition of AchE (46.3% at 10 µM with cpd 5)	[25]
*Aspergillus hiratsukae* SCSIO 5Bn_1_003	Isolation of DM-A3 and 12 other metabolites from this fungus. Its cytotoxic activities against four tumor cell lines (34 < IC_50_ < 50 µM). Inhibition of α-glucosidase (IC_50_ = 35.73 µM) and *Bacillus subtilis* (MIC = 10.26 µg/mL)	[26]
*Aspergillus tennesseensis* 1022LEF	Identification of DM-A3 and characterization of its capacity to inhibit acetylcholinesterase (AchE), NO production, and cytokine expression in activated macrophages.	[27]
*Aspergillus tubingensis* YP-2	Discovery of demethylincisterol A5 from this endophytic fungus and characterization of its cytotoxic properties.	[15]
*Aspergillus* sp.	Isolation of chaxine and (4*S*,17*R*)-4-hydroxy-17-methylincisterol (DM-A3) from a gorgonian-derived fungus, and their antifouling activity.	[28]
*Bipolaris maydis*	Identification of DM-A3 together with 11 new steroids (bipolaristeroids A-K) from this corn pathogenic fungus. Cytotoxic effects of DM-A3 toward different cancer cells in vitro.	[29]
*Cordyceps cicadae*	Identification of 4-hydroxy-17R-methylincisterol (DM-A3) and other metabolites from *C. cicadae* mycelia and spores.	[30]
*Daedaleopsis confragosa*	Identification of DM-A3 and a few other sterols from this fungus. A mild anti-*H. pylori* activity at 100 µM.	[16]
*Diaporthe* sp. LG23	Identification of DM-A3 and volemolide from this fungus on the host plant *Mahonia fortune.*	[31]
*Ganoderma capense* (Lloyd) Teng	Isolation of DM-A3 and its derivative11*α*-hydroxy-21-hydroxy-DM-A3 from the cultured mycelia of this medicinal fungus. No associated bioactivities.	[22]
*Gymnascella dankaliensis*	Identification of DM-A3 and dankastatin C from this fungus.	[18]
*Harposporium anguillulae* YMF 1.01751	Isolation of 7 metabolites including (17*R*)-17-methylincisterol (volemolide) from this endoparasitic nematophagous fungus.	[32]
*Hypholoma lateritium* (Schae.) P. Kumm.	Isolation of DM-A2 and other metabolites from this brick cap mushroom.	[33]
*Nodulisporium* sp. SC-J597	Isolation of DM-A3 and other metabolites from this endophytic fungus. Cytotoxic effects of DM-A3 toward several cancer cell lines in vitro.	[34]
*Penicillium brocae* G2131	Identification of DM-A3 and a closely related compound from this fungus.	[17]
*Penicillium herquei*	Identification of DM-A3 and volemolide from this endophytic fungus to the roots of the Chinese plant *Aconitum carmichaelii*.	[35]
*Penicillium* sp. MCCC 3A00228	Isolation of DM-A3 together with 14 other metabolites from this marine fungus.	[36]
*Pestalotiopsis* sp.	Identification of DM-A3 and ten other metabolites from this fungus endophyte to the Chinese mangrove *Rhizophora mucronata.* Highly potent cytotoxic activity of DM-A3 to HeLa cells (IC_50_ = 0.17 nM).	[37]
*Phyllosticta capitalensis*	An endophyte fungus of the shrub *Loropetalum chinense* var. *rubrum*. Identification of 13 metabolites including demethylincisterol A	[38]
*Plakortis* cfr. *lita*	Isolation of incisterol A2, A5, and A6 from this marine sponge and characterization of the PXR agonist activity of A5 and A6. Binding to the ligand binding domain (LBD) of pregnane X receptor (PXR).	[12]
*Pithomyces sacchari*	Isolation of DM-A3 and characterization of antibacterial effects, notably on QS, biofilm formation, and virulence factors in *B. cereus*.	[39]
*Trichoderma atroviride* RR-dl-3-9	Identification of 4-(p-hydroxyphenethoxy)demethylincisterol A3 from this algicolous fungus. The crystal structure of the compound was determined by X-ray diffraction. Modest anti-phytoplankton and antibacterial effects.	[40]
*Tricholoma imbricatum*	Identification of DM-A3 and volemolide from this fungus. Cytotoxicity against different cancer cell lines.	[19]
*Vanderbylia robiniophila*	Identification of 4-hydroxy-17*R*-methylincisterol (DM-A3) and other metabolites from this medicinal mushroom (*Tametes robiniophila*). No effect on tyrosinase.	[41]
*Xylaria allantoidea* SWUF76	Identification of DM-A3 and chaxine C and characterization of their cytotoxic properties.	[21]
*Xylaria* CGMCC No.5410	An endophytic fungus from the leaves of spikemoss *Selaginella moellendorffii* Hieron. Isolation of DM-A3 and coumarin derivatives	[42]
*Xylaria* sp.	Identification of DM-A3 and other metabolites from this endophytic fungus isolated from traditional Chinese medicinal herb *Coptis chinensis* Franch.	[43,44]

## Abbreviations

The following abbreviations are used in this manuscript:

AchE	Acetylcholinesterase
COX-2	Cyclooxygenase-2
cPGES	Cytosolic prostaglandin E2 synthase
DM-A3	Demethylincisterol A3
IFN-γ	Interferon-γ
IL-2	Interleukin-2
PBMC	Peripheral blood mononuclear cells
PXR	Pregnane X receptor
TNF-α	Tumor necrosis factor-α
